# Increased analgesia administration in emergency medicine after implementation of revised guidelines

**DOI:** 10.1186/s12245-016-0102-y

**Published:** 2016-02-10

**Authors:** Geesje Van Woerden, Crispijn L. Van Den Brand, Cornelis F. Den Hartog, Floris J. Idenburg, Diana C. Grootendorst, M. Christien Van Der Linden

**Affiliations:** Emergency Department, Medical Centre Haaglanden, P.O. Box 432, 2501 CK The Hague, The Netherlands; Department of Anaesthesiology, Medical Centre Haaglanden, P.O. Box 432, 2501 CK The Hague, The Netherlands; Department of Surgery, Medical Centre Haaglanden, P.O. Box 432, 2501 CK The Hague, The Netherlands; Landsteiner Institute, Medical Centre Haaglanden, P.O. Box 432, 2501 CK The Hague, The Netherlands

**Keywords:** Analgesics, Pain management, Acute pain, Emergency department

## Abstract

**Background:**

The most common complaint of patients attending the emergency department (ED) is pain, caused by different diseases. Yet the treatment of pain at the ED is suboptimal, and oligoanalgesia remains common. The objective of this study is to determine whether the administration of analgesia at the ED increases by implementation of revised guidelines in pain management.

**Methods:**

We conducted a prospective pre-post intervention cohort study with implementation of a revised guideline for pain management at our ED, in which nurses are allowed to administer analgesia (including low-dosage piritramid (opioid) intravenous) without doctor intervention. Numeric Rating Scales (NRS) were measured, and administration of medication (main outcome) was documented. We included every adult patient presenting with pain (NRS 4–10) at the ED.

**Results:**

A total of 2107 patients (1089 pre-implementation phase and 1018 post-implementation phase) were included in our study. During pre-implementation, 25.4 % of the patients with NRS between 4 and 10 received analgesia. After implementation, 32.0 % of these patients received analgesia (*p* < 0.001).

**Conclusions:**

After implementation of the revised guidelines in pain management at the ED, the administration of pain medication increased significantly. Nevertheless, the percentage of patients in pain receiving analgesia remain low (32 % after implementation).

## Background

The most common complaint of patients attending the emergency department (ED) is pain, caused by different diseases. The pain prevalence in EDs, throughout the world, ranges from 52 to 79 % [1]. Yet, the treatment of pain at the ED is suboptimal and oligoanalgesia remains common [2]. There are several factors contributing to this oligoanalgesia. First, the Numeric Rating Scale (NRS) to categorise pain as rated by physicians and nurses are both significantly lower than those reported by the patients [3, 4]. Lack of knowledge and lack of guidelines in pain management also contribute to the inadequate treatment of pain [5]. Then, the manner in which a patient expresses pain is partly influenced by personality and culture, which is not always appreciated by a nurse or physician [6]. Lastly, the workload on the ED is high: nurses and physicians are caring for acutely ill patients in addition to patients in pain results in a lack of time to treat the pain properly [7].

### Importance

Thus, improvement of knowledge (among nurses and doctors) and revision of guidelines may increase the administration of analgesia.

Therefore, the objective of the present study is to determine whether the administration of analgesia at the ED increases by implementation of revised guidelines in pain management. Secondly, we will investigate which factors are contributing to pain management at the ED.

## Methods

### Study design

This was a prospective pre-post intervention cohort study with implementation of a revised guideline for pain management to increase the administration of analgesia. The study was divided into two periods (both 1 month) of data collection, before and after implementation of the revised guideline separated by a 1-month interval. During this interval, the revised guideline, developed by the emergency department (emergency physician and nurse) in cooperation with the anaesthesiology and consented by the specialties surgery and internal medicine, was implemented.

### Setting

The study took place in the ED of a level 1 teaching (inner city) hospital and trauma centre with ±50.000 ED visits per year (Medical Centre Haaglanden). The study was conducted between September 2012 and January 2013. Our study was approved by the review board of the Medical Centre Haaglanden and by the Dutch Association of Medical Research Ethics Committee.

### Selection of participants

We included every adult patient (16 years and older) presenting with pain (NRS 4–10) at the ED. As patients entered the ED, nurses assessed patients’ NRS and entered the values into the patients’ charts. Patients with life-threatening diseases/injuries requiring immediate transfer to the operating room or intensive care unit, altered mental status (Glasgow Coma Scale <14) and language barrier were excluded.

### Intervention: revision and implementation of guideline for pain management

A clinical committee was formed, in which hospital staff from the emergency, anaesthesiology and surgical departments were represented. They reviewed the literature and revised the existing guideline for pain management according to the current standard [8]. In comparison to the previous guideline, nurses were allowed to administer analgesia (including low-dosage piritramid (opioid) intravenous (i.v.) without doctor intervention). The revised guideline included the following medication: acetaminophen, NSAID, tramadol, piritramid. Patients with pain NRS >4 were offered pain medication, with higher NRS indicating stronger medication (e.g. patient with NRS = 4–6 were treated initially with acetaminophen per os (p.o.) whereas patient with NRS 7–10 were treated with piritramid i.v.).

**Fig. 1:**
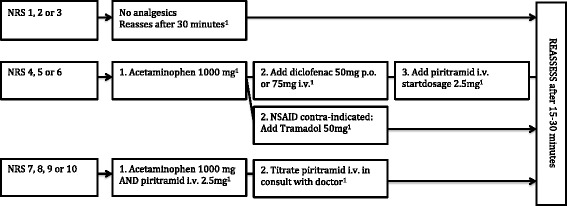
Flowchart pain management. ^1^Reassess after each step, and if NRS is still ≥4, follow next step in the flowchart

The guideline was written in Dutch and distributed to all ED staff and nurses. The ED staff was informed by email. In addition, the ED nurses were also educated face-to-face. Pocket-sized versions (Fig. 1) of the guidelines were distributed, and posters were placed in the ED.

### Methods and measurements

Demographics, pain assessment (NRS) at entrance and leave of the ED, administration of analgesia during ED visit, triage category, medical specialty and usage of analgesia before entering the ED (by patients at home or by EMS personnel) were retrieved from the medical records. Triage levels were assigned according to the five-level Manchester Triage System (MTS) [9]: 1 immediate, 2 very urgent, 3 urgent, 4 standard, and 5 non-urgent. In the analysis, triage levels were clustered because of small numbers of patients with levels 1 and 5, to high (categories 1 and 2), middle (category 3) and low (categories 4 and 5). Triage nurses used the pain ruler of the MTS in obtaining the level of urgency. In addition to the use of the MTS pain ruler, each patient is assessed with the NRS to measure the patients’ actual pain level at entrance and leave of the ED. These NRS scores were used for this study. Medical specialty was divided into two categories: surgical specialty and non-surgical specialty. Surgical specialisms were dermatology, otorhinolaryngology, gynaecology, neurosurgery, ophthalmology, orthopaedics, plastic surgery, surgery, and urology. Non-surgical specialties were cardiology, gastroenterology, internal medicine, neurology, pulmonology, and rheumatology. Patients were managed by emergency physicians as well as by residents. Data collection was conducted from the medical records of the included participants.

### Outcome measures

The main outcome was administration of analgesia in general during ED visit. Secondly, we investigated patient and visit factors that influenced analgesic administration.

### Analysis

Categorical data were expressed as number (%) and compared using the *χ*^2^ test. Continuous data were analysed with a *t* test. A *p* value <0.05 was considered to be significant. The difference in analgesia administration before and after implementation was analysed by multivariate logistic regression analysis, adjusting for the following variables: triage category, usage of analgesia pre-hospital, ethnicity (Dutch versus other ethnicity), gender and age. According to the literature, all these factors are possible confounders in analysing the administration of pain medication [10–18]. We added the variables that were univariately associated with analgesic administration at <0.05. We created our model by the combination of the factors found in literature and the significant factors in our study and adjusted the analysis for these potential confounders. Effect sizes were expressed in adjusted odds ratios (ORs). Statistical uncertainty was expressed in a 95 % confidence interval (CI). Statistical Package for the Social Sciences (SPSS) version 20 was used for all analyses.

## Results

**Fig. 2:**
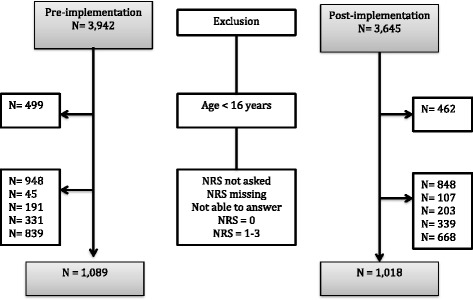
Flow diagram in pre- and post-implementation phases

A total of 2107 patients (1089 resp. 1018) were included in our study (Fig. 2).

### Characteristics of study subjects

Patients enrolled in the two phases of the study were similar with regard to age, gender and ethnicity (Dutch or other). After implementation, there were less patients in the high-NRS group. However, median NRS at admission was comparable between the two time periods. There were differences with regard to the use of analgesia pre-hospital and medical specialty: after implementation, significantly more patients were referred to surgical specialty, and furthermore, the use of analgesia pre-hospital was significantly lower (Table 1).

**Table 1:** Patient characteristics pre- and post-implementation

	Pre-implementation (*n* = 1089)	Post-implementation (*n* = 1018)	*p* value
	Mean years (lowest-highest)	Mean years (lowest-highest)	
Age	42 (16–101)	43 (18–94)	0.86
	Median (std. dev.)	Median (std. dev.)	
NRS at entrance ED (std. dev.)	5.0 (1.7)	5.0 (1.6)	0.086
	*N* (%)	*N* (%)	
Male	526 (48.3)	482 (47.3)	0.66
Dutch	472 (43)	403 (40.0)	0.39
Other	452 (42)	419 (41.2)	
Surgical specialty	386 (35.4)	405 (39.8)	*0.045*
Non-surgical specialty	700 (64.3)	613 (60.2)	
Triage category high	171 (15.7)	153 (15.0)	*0.003*
Triage category middle	509 (46.7)	547 (53.7)	
Triage category low	409 (37.6)	318 (31.2)	
Use of analgesia pre-hospital	150 (13.8)	113 (11.0)	*0.001*
NRS 4–6	698 (64.1)	697 (68.5)	*<0.001*
NRS 7–10	391 (35.9)	321 (31.5)	

P-values printed in italic are statistically significant

### Main results

During the first phase of the study, 25.4 % of the patients with a pain NRS between 4 and 10 received analgesia. After implementation, 31.7 % of the patients in NRS 4–10 received analgesia (*p* = 0.001). The odds to receive analgesia after implementation was 1.35 higher than that before implementation (Table 2). After implementation, patients with a pain NRS between 7 and 10 more often received analgesics than patients with a pain NRS of 4–6 (44 versus 25.7 %, *χ*^2^ < 0.001). After implementation the administration of analgesia was significantly increased in both NRS groups (NRS 4–6: OR 1.41 (1.09–1.83); NRS 7–10: OR 1.43 (1.04–1.96)). There was a significant difference in type of drugs administered after implementation: more acetaminophen was used and combinations of drugs were used more frequently during the post-implementation phase. The route of administration also changed significantly: after implementation, more patients received medication p.o. (comparable with the difference in the administered acetaminophen). There was no difference in the medication given intravenously (Table 3).

**Table 2:** Patient and visit factors that influence analgesic administration

	Crude OR	95 % CI	Adjusted OR*	95 % CI
Implementation of revised guideline for pain management	1.35	1.13–1.65	1.35	1.11–1.65
Triage category				
Low = reference				
Middle	2.40	1.91–3.01	3.00	2.36–3.82
High	2.42	1.79–3.26	3.98	2.86–5.54
Surgical specialty	1.75	1.43–2.15	2.47	1.98–3.09

*Included in the model were medical specialty (surgical versus non-surgical), triage category, usage of analgesia pre-hospital, ethnicity (Dutch versus other ethnicity), gender and age. Only significant variables are shown in the table

**Table 3:** Administration of analgesics: drug types and administration routes

	Pre-implementation	Post-implementation	*p* value
	*N* (%)	*N* (%)	
Administration of analgesics	277 (25.4)	323 (31.7)	*0.001*
Drug types			
Acetaminophen	175 (16.1)	204 (20.0)	*0.018*
NSAID	32 (2.9)	19 (1.9)	0.11
Tramadol	4 (0.4)	4 (0.4)	0.92
Piritramid	5 (0.5)	2 (0.2)	0.30
Drug combination^a^	61 (5.6)	94 (9.2)	*0.001*
Administration routes			
By mouth	171 (15.7)	208 (20.4)	*0.005*
Intravenous	106 (9.7)	115 (11.3)	0.24

^a^Combination of drugs mentioned above (acetaminophen, NSAID, tramadol, piritramid).

P-values printed in italic are statistically significant

In both pre- and post-implementation, several factors contributed to the administration of analgesia. Firstly, the higher triage categories received more frequent analgesia (OR for the highest triage category = 3.98, 95 % CI 2.86–5.54, in which the lowest triage category is the reference category). Secondly, patients treated by a surgical specialty received more frequent analgesia than patients treated by a non-surgical specialty (OR 2.47, 95 % CI 1.98–3.09) (Table 3). This second finding was independent of NRS at entrance ED (OR 2.32, 95 % CI 1.85–2.91; adjusted for variables in Table 2). Age, gender and ethnicity were not significantly correlated to the administration of analgesia.

In a certain number of patients in both groups (before and after implementation), the pain NRS at entrance to the ED was missing, either because it was not established or the patient was unable to answer (Fig. 2). Among these patients, 10 versus 12.7 % (before and after) received analgesics, and more patients were categorised as low urgency by triage compared to the total research population. NRS at leave of the ED was missing in 46 % of the patients (*n* = 973).

## Discussion

This study has several limitations. First, it was a large prospective observational study. Therefore, we can only show associations and no causality. We could not abstract from the charts whether the analgesic administration was protocol driven or by treating physicians. We did not use a blind design because we aimed to modify clinical practice of the ED staff by implementing the revised guidelines. During this period, there were no other changes in pain management (e.g. change in immobilisation, regional blocks), so the effect observed in this pre-post study was likely to be caused by the intervention.

Second, there is a percentage of patients (in both groups) whose pain NRS at entrance to the ED was not measured. Compared to the research group, this group was triaged as less urgent and they received fewer analgesics. We assume that this group was treated during triage and left the ED before being asked the pain NRS. Since the group was triaged as low urgency, the NRS scores are expected to be low (e.g. in case of moderate or severe pain, the triage category is automatically higher), and therefore, it is possible that a large amount of this group would have been excluded (NRS <4). Thus, we assume the effect of our intervention would not change if we had measured the NRS in this group. There are also high urgency patients in this group. An explanation for the missing NRS in this subgroup could be that their injuries/diseases were of such urgency that they had to be transferred to the operation room or intensive care unit immediately. If we had been able to measure the NRS in this high urgency (with expected high NRS scores) category, one could expect the effect of our intervention to be the same or even larger, because analgesic administration is significantly associated with increasing severity of pain [19, 20].

Third, the Hawthorne effect (change of behaviour of nurses and physicians induced by the study itself) is difficult to avoid. Yet, the effect would have been present in both study phases.

Fourth, the use of analgesia pre-hospital was higher before implementation than after implementation. To avoid overdoses, one could expect that less analgesia was administered in the pre-implementation group or more analgesia was administered in the post-implementation group. However, we adjusted the analysis for the use of pre-hospital analgesia and still the difference between the administration of analgesia between pre- and post-implementation was significant.

Fifth, we were not able to measure ethnicity properly, rather than dividing patients into Dutch and not-Dutch based on country of birth of the patient and his or her parents. This may have caused biased information.

Sixth, at baseline, there were differences between the two groups in triage category. Yet, this difference is not explained by more pain, because the median NRS is comparable in the two groups. Although the nurses are obligated to register the administration of medication, it was possible to administer the medication but forget to register this in the patient chart. Thus, the effect size is possibly underestimated.

Finally, the pre- and post-groups differed with respect to specialty and triage category. Therefore, the finding that patients with surgical problems received more often analgesia may have been biased. Still, patients admitted to the ED with a non-surgical problem received less often pain medication than the patients treated by one of the surgical specialties, independent of triage category. Since the education on pain management for all emergency department staff did not differ, this could not explain the found difference. One hypothesis that would explain the difference in specialty in the administration of analgesia would be that tangible proof (e.g. wound, radiographic findings) influences physician/nurse practice more than patients’ reports of pain.

In this study, we demonstrated that after implementation of the revised guideline in pain management at the ED, the administration of pain medication increases significantly. Nevertheless, the percentage of patients in pain receiving analgesia remain low (32 % after implementation). In line with this main outcome, Decosterd et al. [5] showed that the number of patients receiving analgesia increased significantly after implementation of the guidelines. Yet, the increase achieved in their study was more extensive (63 % of the patients received analgesic post-implementation). The smaller effect in our study might be explained by the differences in baseline NRS scores. Though the median NRS was comparable, there were less patients in the post-group with higher NRS scores, possibly leading to less analgesics administration [20].

In our study, inadequate pain management is evident. Several other studies show that around 40 % of patients in pain at the ED do not receive pain medication [21, 22]. This indicates that in this study, oligoanalgesia was and still is a problem. We only measured the administration of analgesics at the ED. The real important issue is whether the pain relief administered has been effective or not [23]. However, in our research, 46 % of these NRS scores at leave were missing, so we were not able to measure effectiveness of administration of analgesics.

To address the differences in pain medication administration, this study found that age was not correlated to the administration of analgesics. Previous research is contradictory: according to Jones et al. [24], elderly patients receive less pain medication whereas a more recent study of Cinar [25] did not find any correlation between age and administration of analgesics. Similar results are known for ethnicity. In our study, we did not find a correlation between ethnicity and administration of analgesics, a finding supported by some literature but refuted by other sources which state that non-white patients are less likely to be treated for pain [10, 12, 14, 17, 18].

According to our study, male and female patients had similar chance of being treated with pain medication. Raftery et al. [26], on the other hand, showed that female patients reported higher pain scores and received more pain medication.

In the future, we would like to test the hypothesis that tangible proof causes differences in the management of pain. There is a high variability in pain management frameworks and protocols among EDs worldwide [27]. The use of our nurse-driven protocol including opioid analgesics based on a pain rating scale has not been validated yet. Since oligoanalgesia is still a major problem in our ED, identifying barriers to ED triage nurses’ management of patients’ pain is important. Also, although less of an issue in The Netherlands, there are concerns of misuse and abuse of opioid analgesics in some other parts of the world [28, 29]. A future study regarding the usage of opioids in Dutch ED triage practice would be of interest. Going forward, we would like to implement a NRS reassessment after the administration of analgesia to measure the effect of the medication. Lastly, a longer-term study into adherence to the new guideline is of interest.

## Conclusions

In summary, we found that after implementation of the revised guidelines in pain management at the ED, the administration of pain medication increased significantly. Nevertheless, the percentage of patients in pain which receive analgesia remain low (32 % after implementation). We also found that triage category and surgical specialty were influencing the administration of pain medication.

## Abbreviations

CI: confidence interval
ED: emergency department
i.v.: intravenous
MTS: Manchester Triage System
NRS: Numeric Rating Scale
OR: odds ratio
p.o.: per os
SPSS: Statistical Package for the Social Sciences
